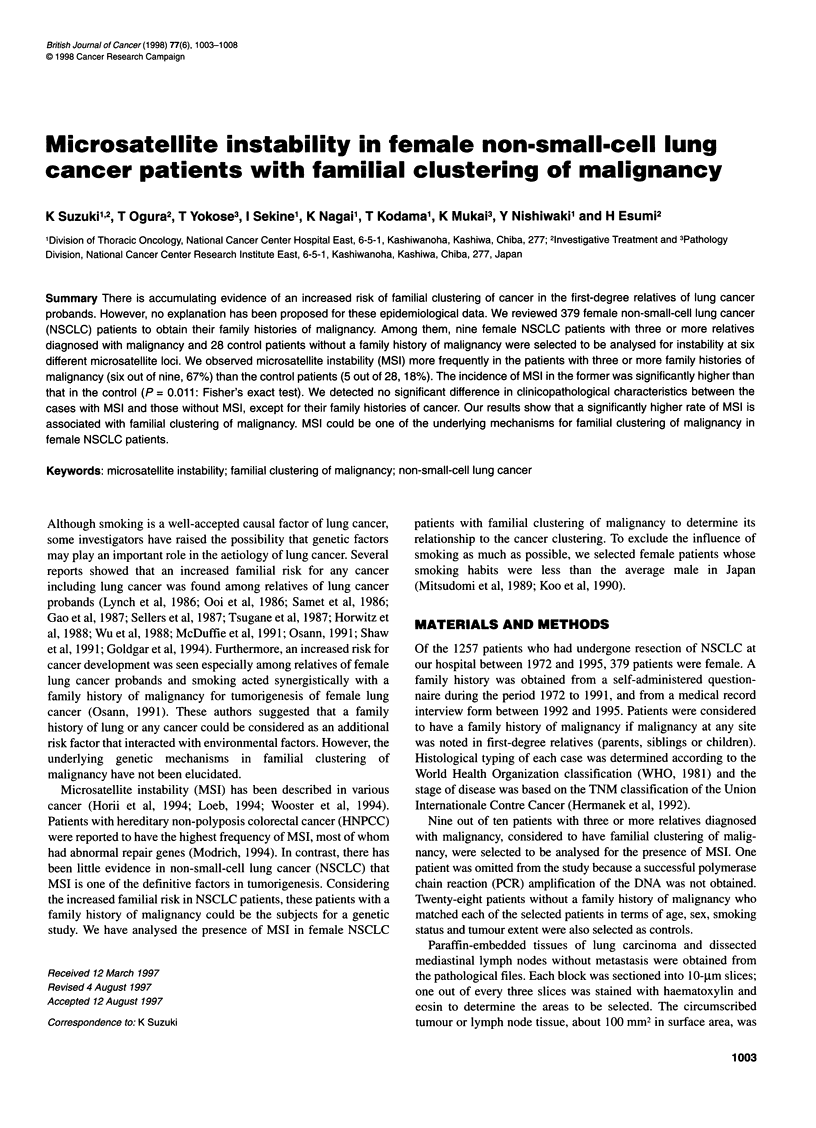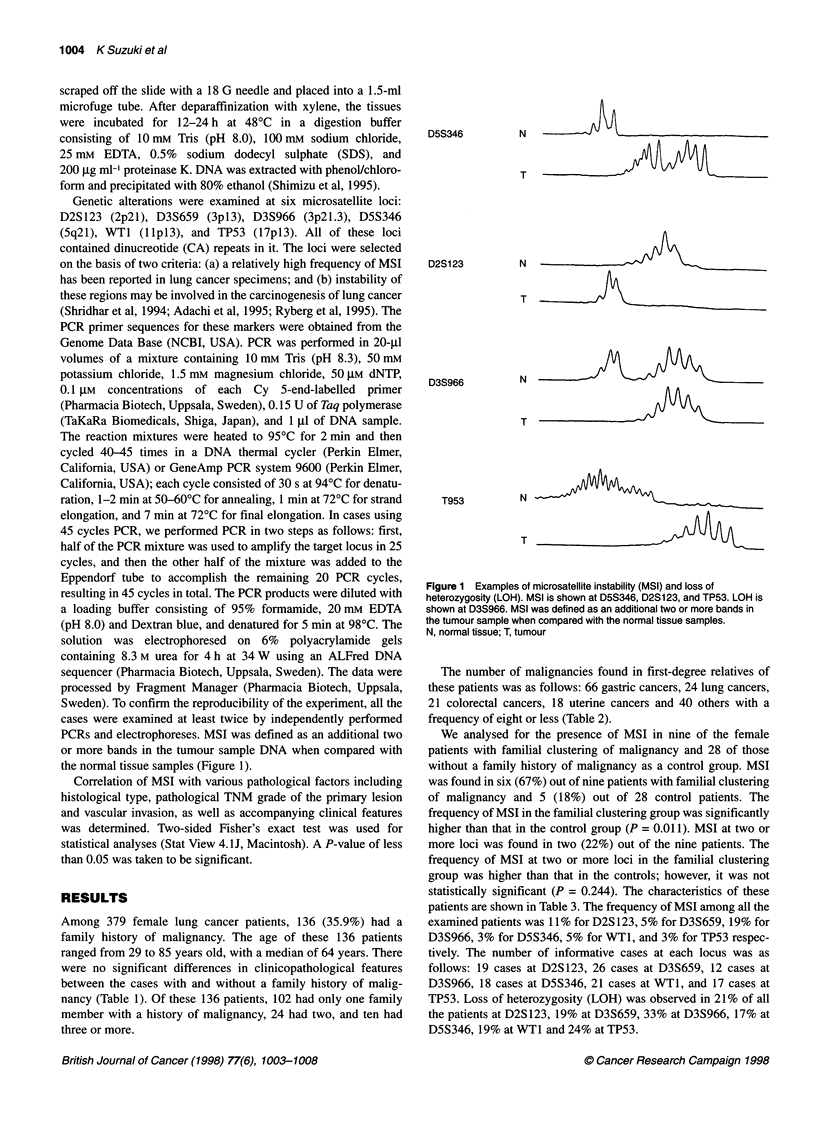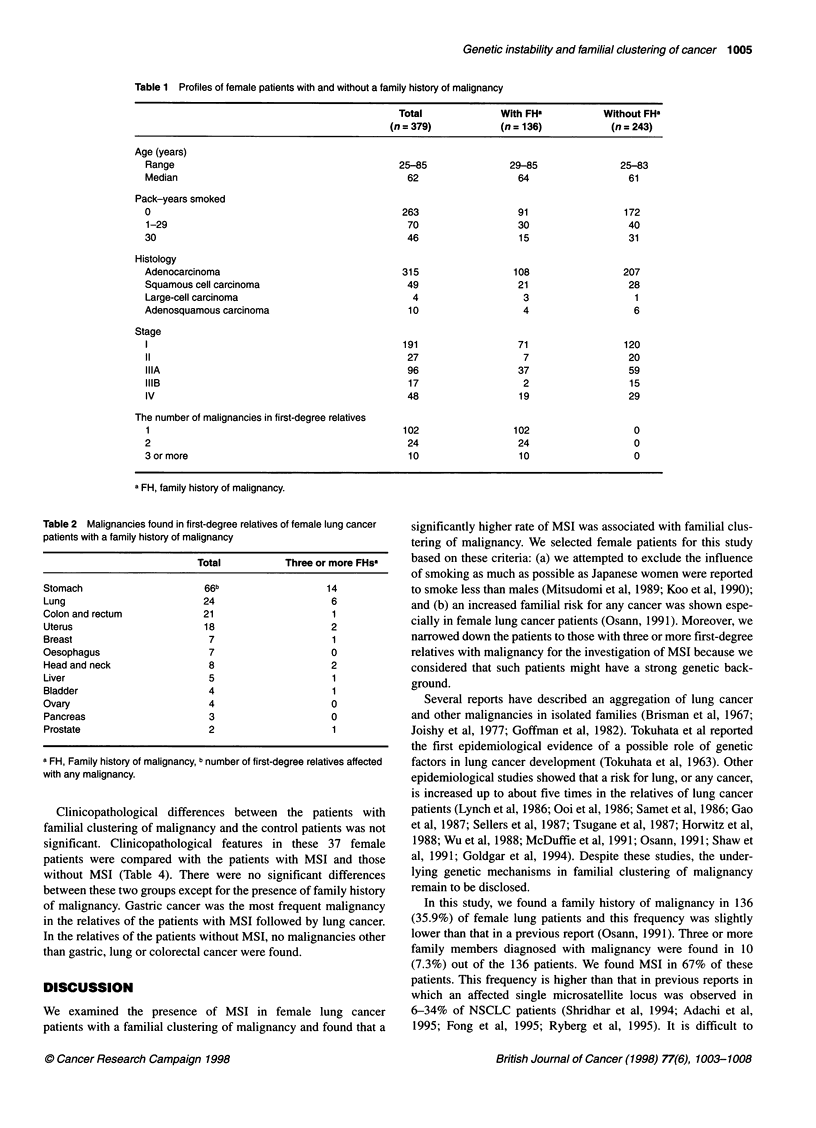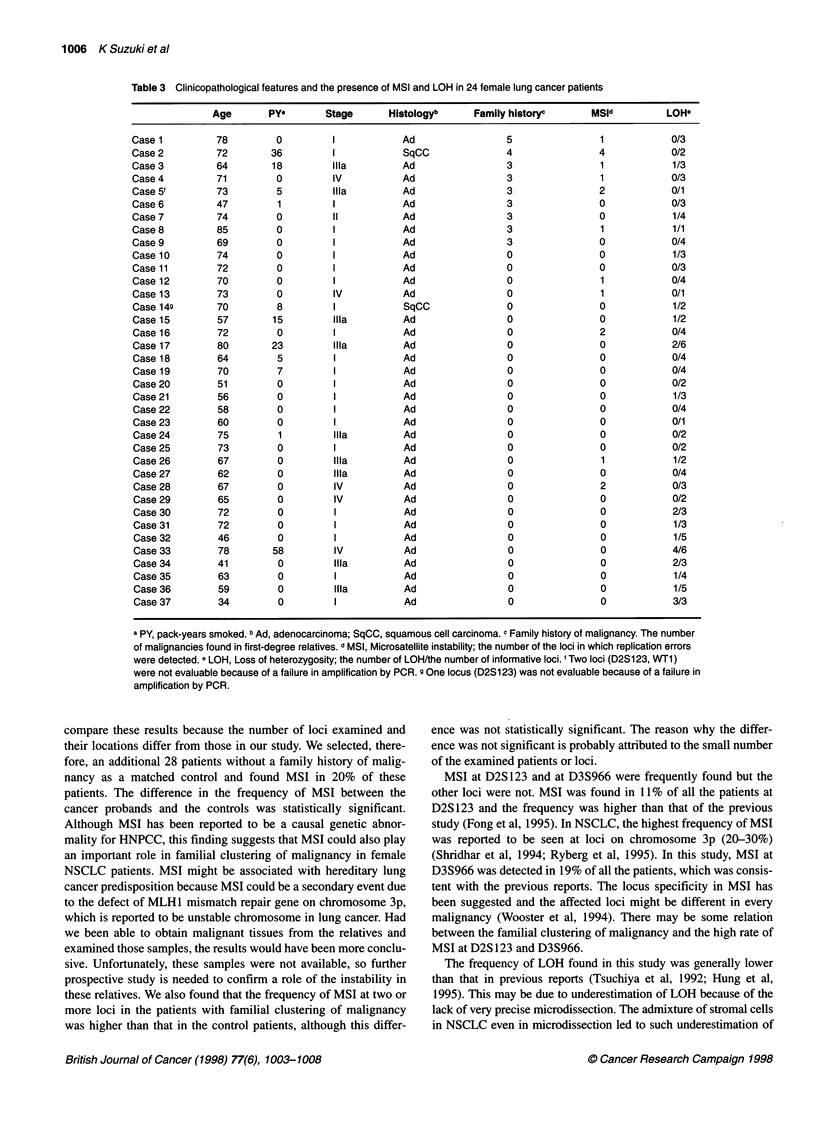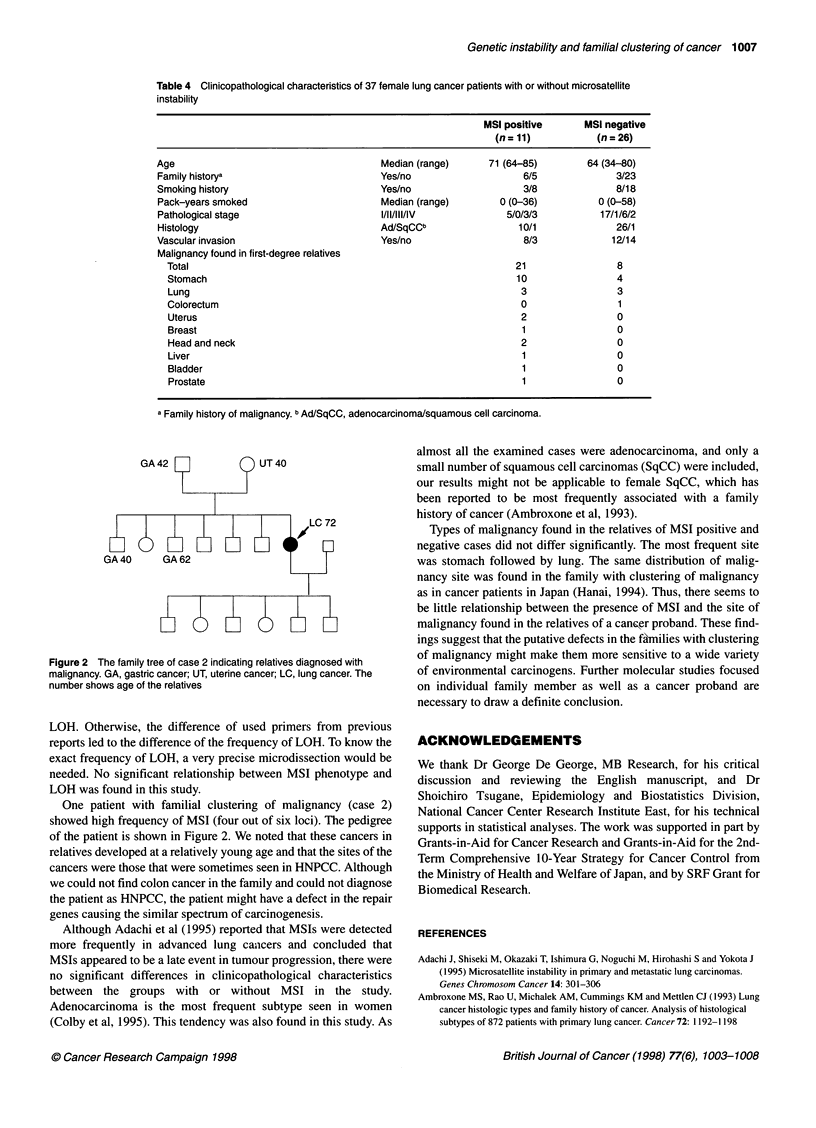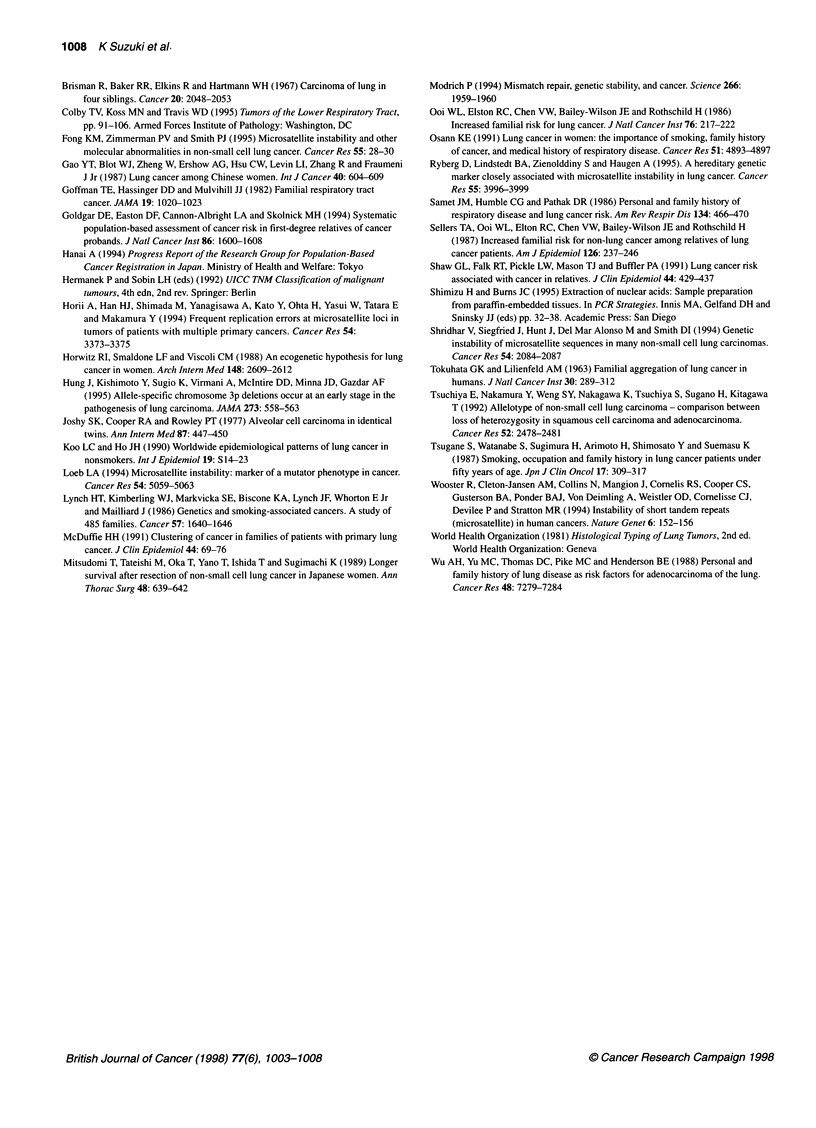# Microsatellite instability in female non-small-cell lung cancer patients with familial clustering of malignancy.

**DOI:** 10.1038/bjc.1998.165

**Published:** 1998-03

**Authors:** K. Suzuki, T. Ogura, T. Yokose, I. Sekine, K. Nagai, T. Kodama, K. Mukai, Y. Nishiwaki, H. Esumi

**Affiliations:** Division of Thoracic Oncology, National Cancer Center Hospital East, Kashiwa, Chiba, Japan.

## Abstract

There is accumulating evidence of an increased risk of familial clustering of cancer in the first-degree relatives of lung cancer probands. However, no explanation has been proposed for these epidemiological data. We reviewed 379 female non-small-cell lung cancer (NSCLC) patients to obtain their family histories of malignancy. Among them, nine female NSCLC patients with three or more relatives diagnosed with malignancy and 28 control patients without a family history of malignancy were selected to be analysed for instability at six different microsatellite loci. We observed microsatellite instability (MSI) more frequently in the patients with three or more family histories of malignancy (six out of nine, 67%) than the control patients (5 out of 28, 18%). The incidence of MSI in the former was significantly higher than that in the control (P=0.011: Fisher's exact test). We detected no significant difference in clinicopathological characteristics between the cases with MSI and those without MSI, except for their family histories of cancer. Our results show that a significantly higher rate of MSI is associated with familial clustering of malignancy. MSI could be one of the underlying mechanisms for familial clustering of malignancy in female NSCLC patients.


British Journal of Cancer (1998) 77(6), 1003–1008
© 1998 Cancer Research Campaign

### Microsatellite instability in female non-small-cell lung cancer patients with familial clustering of malignancy

K Suzuki[1,2], T Ogura[2], T Yokose[3], I Sekine[1], K Nagai[1], T Kodama[1], K Mukai[3], Y Nishiwaki[1] and H Esumi[2]

[1]Division of Thoracic Oncology, National Cancer Center Hospital East, 6-5-1, Kashiwanoha, Kashiwa, Chiba, 277; [2]Investigative Treatment and [3]Pathology Division, National Cancer Center Research Institute East, 6-5-1, Kashiwanoha, Kashiwa, Chiba, 277, Japan

**Summary** There is accumulating evidence of an increased risk of familial clustering of cancer in the first-degree relatives of lung cancer probands. However, no explanation has been proposed for these epidemiological data. We reviewed 379 female non-small-cell lung cancer (NSCLC) patients to obtain their family histories of malignancy. Among them, nine female NSCLC patients with three or more relatives diagnosed with malignancy and 28 control patients without a family history of malignancy were selected to be analysed for instability at six different microsatellite loci. We observed microsatellite instability (MSI) more frequently in the patients with three or more family histories of malignancy (six out of nine, 67%) than the control patients (5 out of 28, 18%). The incidence of MSI in the former was significantly higher than that in the control ($P = 0.011$: Fisher's exact test). We detected no significant difference in clinicopathological characteristics between the cases with MSI and those without MSI, except for their family histories of cancer. Our results show that a significantly higher rate of MSI is associated with familial clustering of malignancy. MSI could be one of the underlying mechanisms for familial clustering of malignancy in female NSCLC patients.

**Keywords**: microsatellite instability; familial clustering of malignancy; non-small-cell lung cancer

Although smoking is a well-accepted causal factor of lung cancer, some investigators have raised the possibility that genetic factors may play an important role in the aetiology of lung cancer. Several reports showed that an increased familial risk for any cancer including lung cancer was found among relatives of lung cancer probands (Lynch et al, 1986; Ooi et al, 1986; Samet et al, 1986; Gao et al, 1987; Sellers et al, 1987; Tsugane et al, 1987; Horwitz et al, 1988; Wu et al, 1988; McDuffie et al, 1991; Osann, 1991; Shaw et al, 1991; Goldgar et al, 1994). Furthermore, an increased risk for cancer development was seen especially among relatives of female lung cancer probands and smoking acted synergistically with a family history of malignancy for tumorigenesis of female lung cancer (Osann, 1991). These authors suggested that a family history of lung or any cancer could be considered as an additional risk factor that interacted with environmental factors. However, the underlying genetic mechanisms in familial clustering of malignancy have not been elucidated.

Microsatellite instability (MSI) has been described in various cancer (Horii et al, 1994; Loeb, 1994; Wooster et al, 1994). Patients with hereditary non-polyposis colorectal cancer (HNPCC) were reported to have the highest frequency of MSI, most of whom had abnormal repair genes (Modrich, 1994). In contrast, there has been little evidence in non-small-cell lung cancer (NSCLC) that MSI is one of the definitive factors in tumorigenesis. Considering the increased familial risk in NSCLC patients, these patients with a family history of malignancy could be the subjects for a genetic study. We have analysed the presence of MSI in female NSCLC patients with familial clustering of malignancy to determine its relationship to the cancer clustering. To exclude the influence of smoking as much as possible, we selected female patients whose smoking habits were less than the average male in Japan (Mitsudomi et al, 1989; Koo et al, 1990).

*Received 12 March 1997*
*Revised 4 August 1997*
*Accepted 12 August 1997*

*Correspondence to:* K Suzuki

### MATERIALS AND METHODS

Of the 1257 patients who had undergone resection of NSCLC at our hospital between 1972 and 1995, 379 patients were female. A family history was obtained from a self-administered questionnaire during the period 1972 to 1991, and from a medical record interview form between 1992 and 1995. Patients were considered to have a family history of malignancy if malignancy at any site was noted in first-degree relatives (parents, siblings or children). Histological typing of each case was determined according to the World Health Organization classification (WHO, 1981) and the stage of disease was based on the TNM classification of the Union Internationale Contre Cancer (Hermanek et al, 1992).

Nine out of ten patients with three or more relatives diagnosed with malignancy, considered to have familial clustering of malignancy, were selected to be analysed for the presence of MSI. One patient was omitted from the study because a successful polymerase chain reaction (PCR) amplification of the DNA was not obtained. Twenty-eight patients without a family history of malignancy who matched each of the selected patients in terms of age, sex, smoking status and tumour extent were also selected as controls.

Paraffin-embedded tissues of lung carcinoma and dissected mediastinal lymph nodes without metastasis were obtained from the pathological files. Each block was sectioned into 10-μm slices; one out of every three slices was stained with haematoxylin and eosin to determine the areas to be selected. The circumscribed tumour or lymph node tissue, about 100 $mm^2$ in surface area, was scraped off the slide with a 18 G needle and placed into a 1.5-ml microfuge tube. After deparaffinization with xylene, the tissues were incubated for 12–24 h at 48°C in a digestion buffer consisting of 10 mM Tris (pH 8.0), 100 mM sodium chloride, 25 mM EDTA, 0.5% sodium dodecyl sulphate (SDS), and 200 μg $ml^{-1}$ proteinase K. DNA was extracted with phenol/chloroform and precipitated with 80% ethanol (Shimizu et al, 1995).

Genetic alterations were examined at six microsatellite loci: D2S123 (2p21), D3S659 (3p13), D3S966 (3p21.3), D5S346 (5q21), WT1 (11p13), and TP53 (17p13). All of these loci contained dinucreotide (CA) repeats in it. The loci were selected on the basis of two criteria: (a) a relatively high frequency of MSI has been reported in lung cancer specimens; and (b) instability of these regions may be involved in the carcinogenesis of lung cancer (Shridhar et al, 1994; Adachi et al, 1995; Ryberg et al, 1995). The PCR primer sequences for these markers were obtained from the Genome Data Base (NCBI, USA). PCR was performed in 20-μl volumes of a mixture containing 10 mM Tris (pH 8.3), 50 mM potassium chloride, 1.5 mM magnesium chloride, 50 μM dNTP, 0.1 μM concentrations of each Cy 5-end-labelled primer (Pharmacia Biotech, Uppsala, Sweden), 0.15 U of *Taq* polymerase (TaKaRa Biomedicals, Shiga, Japan), and 1 μl of DNA sample. The reaction mixtures were heated to 95°C for 2 min and then cycled 40–45 times in a DNA thermal cycler (Perkin Elmer, California, USA) or GeneAmp PCR system 9600 (Perkin Elmer, California, USA); each cycle consisted of 30 s at 94°C for denaturation, 1–2 min at 50–60°C for annealing, 1 min at 72°C for strand elongation, and 7 min at 72°C for final elongation. In cases using 45 cycles PCR, we performed PCR in two steps as follows: first, half of the PCR mixture was used to amplify the target locus in 25 cycles, and then the other half of the mixture was added to the Eppendorf tube to accomplish the remaining 20 PCR cycles, resulting in 45 cycles in total. The PCR products were diluted with a loading buffer consisting of 95% formamide, 20 mM EDTA (pH 8.0) and Dextran blue, and denatured for 5 min at 98°C. The solution was electrophoresed on 6% polyacrylamide gels containing 8.3 M urea for 4 h at 34 W using an ALFred DNA sequencer (Pharmacia Biotech, Uppsala, Sweden). The data were processed by Fragment Manager (Pharmacia Biotech, Uppsala, Sweden). To confirm the reproducibility of the experiment, all the cases were examined at least twice by independently performed PCRs and electrophoreses. MSI was defined as an additional two or more bands in the tumour sample DNA when compared with the normal tissue samples (Figure 1).

Correlation of MSI with various pathological factors including histological type, pathological TNM grade of the primary lesion and vascular invasion, as well as accompanying clinical features was determined. Two-sided Fisher's exact test was used for statistical analyses (Stat View 4.1J, Macintosh). A *P*-value of less than 0.05 was taken to be significant.

### RESULTS

Among 379 female lung cancer patients, 136 (35.9%) had a family history of malignancy. The age of these 136 patients ranged from 29 to 85 years old, with a median of 64 years. There were no significant differences in clinicopathological features between the cases with and without a family history of malignancy (Table 1). Of these 136 patients, 102 had only one family member with a history of malignancy, 24 had two, and ten had three or more.

**Figure 1** Examples of microsatellite instability (MSI) and loss of heterozygosity (LOH). MSI is shown at D5S346, D2S123, and TP53. LOH is shown at D3S966. MSI was defined as an additional two or more bands in the tumour sample when compared with the normal tissue samples.
N, normal tissue; T, tumour

The number of malignancies found in first-degree relatives of these patients was as follows: 66 gastric cancers, 24 lung cancers, 21 colorectal cancers, 18 uterine cancers and 40 others with a frequency of eight or less (Table 2).

We analysed for the presence of MSI in nine of the female patients with familial clustering of malignancy and 28 of those without a family history of malignancy as a control group. MSI was found in six (67%) out of nine patients with familial clustering of malignancy and 5 (18%) out of 28 control patients. The frequency of MSI in the familial clustering group was significantly higher than that in the control group ($P$ = 0.011). MSI at two or more loci was found in two (22%) out of the nine patients. The frequency of MSI at two or more loci in the familial clustering group was higher than that in the controls; however, it was not statistically significant ($P$ = 0.244). The characteristics of these patients are shown in Table 3. The frequency of MSI among all the examined patients was 11% for D2S123, 5% for D3S659, 19% for D3S966, 3% for D5S346, 5% for WT1, and 3% for TP53 respectively. The number of informative cases at each locus was as follows: 19 cases at D2S123, 26 cases at D3S659, 12 cases at D3S966, 18 cases at D5S346, 21 cases at WT1, and 17 cases at TP53. Loss of heterozygosity (LOH) was observed in 21% of all the patients at D2S123, 19% at D3S659, 33% at D3S966, 17% at D5S346, 19% at WT1 and 24% at TP53.

© Cancer Research Campaign 1998

**Table 1** Profiles of female patients with and without a family history of malignancy

| | Total ($n$ = 379) | With FH[a] ($n$ = 136) | Without FH[a] ($n$ = 243) |
|---|---|---|---|
| Age (years) | | | |
| Range | 25–85 | 29–85 | 25–83 |
| Median | 62 | 64 | 61 |
| Pack–years smoked | | | |
| 0 | 263 | 91 | 172 |
| 1–29 | 70 | 30 | 40 |
| 30 | 46 | 15 | 31 |
| Histology | | | |
| Adenocarcinoma | 315 | 108 | 207 |
| Squamous cell carcinoma | 49 | 21 | 28 |
| Large-cell carcinoma | 4 | 3 | 1 |
| Adenosquamous carcinoma | 10 | 4 | 6 |
| Stage | | | |
| I | 191 | 71 | 120 |
| II | 27 | 7 | 20 |
| IIIA | 96 | 37 | 59 |
| IIIB | 17 | 2 | 15 |
| IV | 48 | 19 | 29 |
| The number of malignancies in first-degree relatives | | | |
| 1 | 102 | 102 | 0 |
| 2 | 24 | 24 | 0 |
| 3 or more | 10 | 10 | 0 |

[a] FH, family history of malignancy.

**Table 2** Malignancies found in first-degree relatives of female lung cancer patients with a family history of malignancy

| | Total | Three or more FHs[a] |
|---|---|---|
| Stomach | 66[b] | 14 |
| Lung | 24 | 6 |
| Colon and rectum | 21 | 1 |
| Uterus | 18 | 2 |
| Breast | 7 | 1 |
| Oesophagus | 7 | 0 |
| Head and neck | 8 | 2 |
| Liver | 5 | 1 |
| Bladder | 4 | 1 |
| Ovary | 4 | 0 |
| Pancreas | 3 | 0 |
| Prostate | 2 | 1 |

[a] FH, Family history of malignancy, [b] number of first-degree relatives affected with any malignancy.

Clinicopathological differences between the patients with familial clustering of malignancy and the control patients was not significant. Clinicopathological features in these 37 female patients were compared with the patients with MSI and those without MSI (Table 4). There were no significant differences between these two groups except for the presence of family history of malignancy. Gastric cancer was the most frequent malignancy in the relatives of the patients with MSI followed by lung cancer. In the relatives of the patients without MSI, no malignancies other than gastric, lung or colorectal cancer were found.

### DISCUSSION

We examined the presence of MSI in female lung cancer patients with a familial clustering of malignancy and found that a significantly higher rate of MSI was associated with familial clustering of malignancy. We selected female patients for this study based on these criteria: (a) we attempted to exclude the influence of smoking as much as possible as Japanese women were reported to smoke less than males (Mitsudomi et al, 1989; Koo et al, 1990); and (b) an increased familial risk for any cancer was shown especially in female lung cancer patients (Osann, 1991). Moreover, we narrowed down the patients to those with three or more first-degree relatives with malignancy for the investigation of MSI because we considered that such patients might have a strong genetic background.

Several reports have described an aggregation of lung cancer and other malignancies in isolated families (Brisman et al, 1967; Joishy et al, 1977; Goffman et al, 1982). Tokuhata et al reported the first epidemiological evidence of a possible role of genetic factors in lung cancer development (Tokuhata et al, 1963). Other epidemiological studies showed that a risk for lung, or any cancer, is increased up to about five times in the relatives of lung cancer patients (Lynch et al, 1986; Ooi et al, 1986; Samet et al, 1986; Gao et al, 1987; Sellers et al, 1987; Tsugane et al, 1987; Horwitz et al, 1988; Wu et al, 1988; McDuffie et al, 1991; Osann, 1991; Shaw et al, 1991; Goldgar et al, 1994). Despite these studies, the underlying genetic mechanisms in familial clustering of malignancy remain to be disclosed.

In this study, we found a family history of malignancy in 136 (35.9%) of female lung patients and this frequency was slightly lower than that in a previous report (Osann, 1991). Three or more family members diagnosed with malignancy were found in 10 (7.3%) out of the 136 patients. We found MSI in 67% of these patients. This frequency is higher than that in previous reports in which an affected single microsatellite locus was observed in 6–34% of NSCLC patients (Shridhar et al, 1994; Adachi et al, 1995; Fong et al, 1995; Ryberg et al, 1995). It is difficult to

© Cancer Research Campaign 1998

Table 3 Clinicopathological features and the presence of MSI and LOH in 24 female lung cancer patients

| | Age | PY[a] | Stage | Histology[b] | Family history[c] | MSI[d] | LOH[e] |
|---|---|---|---|---|---|---|---|
| Case 1 | 78 | 0 | I | Ad | 5 | 1 | 0/3 |
| Case 2 | 72 | 36 | I | SqCC | 4 | 4 | 0/2 |
| Case 3 | 64 | 18 | IIIa | Ad | 3 | 1 | 1/3 |
| Case 4 | 71 | 0 | IV | Ad | 3 | 1 | 0/3 |
| Case 5[f] | 73 | 5 | IIIa | Ad | 3 | 2 | 0/1 |
| Case 6 | 47 | 1 | I | Ad | 3 | 0 | 0/3 |
| Case 7 | 74 | 0 | II | Ad | 3 | 0 | 1/4 |
| Case 8 | 85 | 0 | I | Ad | 3 | 1 | 1/1 |
| Case 9 | 69 | 0 | I | Ad | 3 | 0 | 0/4 |
| Case 10 | 74 | 0 | I | Ad | 0 | 0 | 1/3 |
| Case 11 | 72 | 0 | I | Ad | 0 | 0 | 0/3 |
| Case 12 | 70 | 0 | I | Ad | 0 | 1 | 0/4 |
| Case 13 | 73 | 0 | IV | Ad | 0 | 1 | 0/1 |
| Case 14[g] | 70 | 8 | I | SqCC | 0 | 0 | 1/2 |
| Case 15 | 57 | 15 | IIIa | Ad | 0 | 0 | 1/2 |
| Case 16 | 72 | 0 | I | Ad | 0 | 2 | 0/4 |
| Case 17 | 80 | 23 | IIIa | Ad | 0 | 0 | 2/6 |
| Case 18 | 64 | 5 | I | Ad | 0 | 0 | 0/4 |
| Case 19 | 70 | 7 | I | Ad | 0 | 0 | 0/4 |
| Case 20 | 51 | 0 | I | Ad | 0 | 0 | 0/2 |
| Case 21 | 56 | 0 | I | Ad | 0 | 0 | 1/3 |
| Case 22 | 58 | 0 | I | Ad | 0 | 0 | 0/4 |
| Case 23 | 60 | 0 | I | Ad | 0 | 0 | 0/1 |
| Case 24 | 75 | 1 | IIIa | Ad | 0 | 0 | 0/2 |
| Case 25 | 73 | 0 | I | Ad | 0 | 0 | 0/2 |
| Case 26 | 67 | 0 | IIIa | Ad | 0 | 1 | 1/2 |
| Case 27 | 62 | 0 | IIIa | Ad | 0 | 0 | 0/4 |
| Case 28 | 67 | 0 | IV | Ad | 0 | 2 | 0/3 |
| Case 29 | 65 | 0 | IV | Ad | 0 | 0 | 0/2 |
| Case 30 | 72 | 0 | I | Ad | 0 | 0 | 2/3 |
| Case 31 | 72 | 0 | I | Ad | 0 | 0 | 1/3 |
| Case 32 | 46 | 0 | I | Ad | 0 | 0 | 1/5 |
| Case 33 | 78 | 58 | IV | Ad | 0 | 0 | 4/6 |
| Case 34 | 41 | 0 | IIIa | Ad | 0 | 0 | 2/3 |
| Case 35 | 63 | 0 | I | Ad | 0 | 0 | 1/4 |
| Case 36 | 59 | 0 | IIIa | Ad | 0 | 0 | 1/5 |
| Case 37 | 34 | 0 | I | Ad | 0 | 0 | 3/3 |

[a] PY, pack-years smoked. [b] Ad, adenocarcinoma; SqCC, squamous cell carcinoma. [c] Family history of malignancy. The number of malignancies found in first-degree relatives. [d] MSI, Microsatellite instability; the number of the loci in which replication errors were detected. [e] LOH, Loss of heterozygosity; the number of LOH/the number of informative loci. [f] Two loci (D2S123, WT1) were not evaluable because of a failure in amplification by PCR. [g] One locus (D2S123) was not evaluable because of a failure in amplification by PCR.

compare these results because the number of loci examined and their locations differ from those in our study. We selected, therefore, an additional 28 patients without a family history of malignancy as a matched control and found MSI in 20% of these patients. The difference in the frequency of MSI between the cancer probands and the controls was statistically significant. Although MSI has been reported to be a causal genetic abnormality for HNPCC, this finding suggests that MSI could also play an important role in familial clustering of malignancy in female NSCLC patients. MSI might be associated with hereditary lung cancer predisposition because MSI could be a secondary event due to the defect of MLH1 mismatch repair gene on chromosome 3p, which is reported to be unstable chromosome in lung cancer. Had we been able to obtain malignant tissues from the relatives and examined those samples, the results would have been more conclusive. Unfortunately, these samples were not available, so further prospective study is needed to confirm a role of the instability in these relatives. We also found that the frequency of MSI at two or more loci in the patients with familial clustering of malignancy was higher than that in the control patients, although this difference was not statistically significant. The reason why the difference was not significant is probably attributed to the small number of the examined patients or loci.

MSI at D2S123 and at D3S966 were frequently found but the other loci were not. MSI was found in 11% of all the patients at D2S123 and the frequency was higher than that of the previous study (Fong et al, 1995). In NSCLC, the highest frequency of MSI was reported to be seen at loci on chromosome 3p (20–30%) (Shridhar et al, 1994; Ryberg et al, 1995). In this study, MSI at D3S966 was detected in 19% of all the patients, which was consistent with the previous reports. The locus specificity in MSI has been suggested and the affected loci might be different in every malignancy (Wooster et al, 1994). There may be some relation between the familial clustering of malignancy and the high rate of MSI at D2S123 and D3S966.

The frequency of LOH found in this study was generally lower than that in previous reports (Tsuchiya et al, 1992; Hung et al, 1995). This may be due to underestimation of LOH because of the lack of very precise microdissection. The admixture of stromal cells in NSCLC even in microdissection led to such underestimation of

 © Cancer Research Campaign 1998

**Table 4** Clinicopathological characteristics of 37 female lung cancer patients with or without microsatellite instability

| | | **MSI positive ($n$ = 11)** | **MSI negative ($n$ = 26)** |
|---|---|---|---|
| Age | Median (range) | 71 (64–85) | 64 (34–80) |
| Family history[a] | Yes/no | 6/5 | 3/23 |
| Smoking history | Yes/no | 3/8 | 8/18 |
| Pack–years smoked | Median (range) | 0 (0–36) | 0 (0–58) |
| Pathological stage | I/II/III/IV | 5/0/3/3 | 17/1/6/2 |
| Histology | Ad/SqCC[b] | 10/1 | 26/1 |
| Vascular invasion | Yes/no | 8/3 | 12/14 |
| Malignancy found in first-degree relatives | | | |
| Total | | 21 | 8 |
| Stomach | | 10 | 4 |
| Lung | | 3 | 3 |
| Colorectum | | 0 | 1 |
| Uterus | | 2 | 0 |
| Breast | | 1 | 0 |
| Head and neck | | 2 | 0 |
| Liver | | 1 | 0 |
| Bladder | | 1 | 0 |
| Prostate | | 1 | 0 |

[a] Family history of malignancy. [b] Ad/SqCC, adenocarcinoma/squamous cell carcinoma.

**Figure 2** The family tree of case 2 indicating relatives diagnosed with malignancy. GA, gastric cancer; UT, uterine cancer; LC, lung cancer. The number shows age of the relatives

LOH. Otherwise, the difference of used primers from previous reports led to the difference of the frequency of LOH. To know the exact frequency of LOH, a very precise microdissection would be needed. No significant relationship between MSI phenotype and LOH was found in this study.

One patient with familial clustering of malignancy (case 2) showed high frequency of MSI (four out of six loci). The pedigree of the patient is shown in Figure 2. We noted that these cancers in relatives developed at a relatively young age and that the sites of the cancers were those that were sometimes seen in HNPCC. Although we could not find colon cancer in the family and could not diagnose the patient as HNPCC, the patient might have a defect in the repair genes causing the similar spectrum of carcinogenesis.

Although Adachi et al (1995) reported that MSIs were detected more frequently in advanced lung cancers and concluded that MSIs appeared to be a late event in tumour progression, there were no significant differences in clinicopathological characteristics between the groups with or without MSI in the study. Adenocarcinoma is the most frequent subtype seen in women (Colby et al, 1995). This tendency was also found in this study. As almost all the examined cases were adenocarcinoma, and only a small number of squamous cell carcinomas (SqCC) were included, our results might not be applicable to female SqCC, which has been reported to be most frequently associated with a family history of cancer (Ambroxone et al, 1993).

Types of malignancy found in the relatives of MSI positive and negative cases did not differ significantly. The most frequent site was stomach followed by lung. The same distribution of malignancy site was found in the family with clustering of malignancy as in cancer patients in Japan (Hanai, 1994). Thus, there seems to be little relationship between the presence of MSI and the site of malignancy found in the relatives of a cancer proband. These findings suggest that the putative defects in the families with clustering of malignancy might make them more sensitive to a wide variety of environmental carcinogens. Further molecular studies focused on individual family member as well as a cancer proband are necessary to draw a definite conclusion.

### ACKNOWLEDGEMENTS

We thank Dr George De George, MB Research, for his critical discussion and reviewing the English manuscript, and Dr Shoichiro Tsugane, Epidemiology and Biostatistics Division, National Cancer Center Research Institute East, for his technical supports in statistical analyses. The work was supported in part by Grants-in-Aid for Cancer Research and Grants-in-Aid for the 2nd-Term Comprehensive 10-Year Strategy for Cancer Control from the Ministry of Health and Welfare of Japan, and by SRF Grant for Biomedical Research.

### REFERENCES

Adachi J, Shiseki M, Okazaki T, Ishimura G, Noguchi M, Hirohashi S and Yokota J (1995) Microsatellite instability in primary and metastatic lung carcinomas. *Genes Chromosom Cancer* **14**: 301–306

Ambroxone MS, Rao U, Michalek AM, Cummings KM and Mettlen CJ (1993) Lung cancer histologic types and family history of cancer. Analysis of histological subtypes of 872 patients with primary lung cancer. *Cancer* **72**: 1192–1198

© Cancer Research Campaign 1998

Brisman R, Baker RR, Elkins R and Hartmann WH (1967) Carcinoma of lung in four siblings. *Cancer* **20**: 2048–2053

Colby TV, Koss MN and Travis WD (1995) *Tumors of the Lower Respiratory Tract*, pp. 91–106. Armed Forces Institute of Pathology: Washington, DC

Fong KM, Zimmerman PV and Smith PJ (1995) Microsatellite instability and other molecular abnormalities in non-small cell lung cancer. *Cancer Res* **55**: 28–30

Gao YT, Blot WJ, Zheng W, Ershow AG, Hsu CW, Levin LI, Zhang R and Fraumeni J Jr (1987) Lung cancer among Chinese women. *Int J Cancer* **40**: 604–609

Goffman TE, Hassinger DD and Mulvihill JJ (1982) Familial respiratory tract cancer. *JAMA* **19**: 1020–1023

Goldgar DE, Easton DF, Cannon-Albright LA and Skolnick MH (1994) Systematic population-based assessment of cancer risk in first-degree relatives of cancer probands. *J Natl Cancer Inst* **86**: 1600–1608

Hanai A (1994) *Progress Report of the Research Group for Population-Based Cancer Registration in Japan*. Ministry of Health and Welfare: Tokyo

Hermanek P and Sobin LH (eds) (1992) *UICC TNM Classification of malignant tumours*, 4th edn, 2nd rev. Springer: Berlin

Horii A, Han HJ, Shimada M, Yanagisawa A, Kato Y, Ohta H, Yasui W, Tatara E and Makamura Y (1994) Frequent replication errors at microsatellite loci in tumors of patients with multiple primary cancers. *Cancer Res* **54**: 3373–3375

Horwitz RI, Smaldone LF and Viscoli CM (1988) An ecogenetic hypothesis for lung cancer in women. *Arch Intern Med* **148**: 2609–2612

Hung J, Kishimoto Y, Sugio K, Virmani A, McIntire DD, Minna JD, Gazdar AF (1995) Allele-specific chromosome 3p deletions occur at an early stage in the pathogenesis of lung carcinoma. *JAMA* **273**: 558–563

Joshy SK, Cooper RA and Rowley PT (1977) Alveolar cell carcinoma in identical twins. *Ann Intern Med* **87**: 447–450

Koo LC and Ho JH (1990) Worldwide epidemiological patterns of lung cancer in nonsmokers. *Int J Epidemiol* **19**: S14–23

Loeb LA (1994) Microsatellite instability: marker of a mutator phenotype in cancer. *Cancer Res* **54**: 5059–5063

Lynch HT, Kimberling WJ, Markvicka SE, Biscone KA, Lynch JF, Whorton E Jr and Mailliard J (1986) Genetics and smoking-associated cancers. A study of 485 families. *Cancer* **57**: 1640–1646

McDuffie HH (1991) Clustering of cancer in families of patients with primary lung cancer. *J Clin Epidemiol* **44**: 69–76

Mitsudomi T, Tateishi M, Oka T, Yano T, Ishida T and Sugimachi K (1989) Longer survival after resection of non-small cell lung cancer in Japanese women. *Ann Thorac Surg* **48**: 639–642

Modrich P (1994) Mismatch repair, genetic stability, and cancer. *Science* **266**: 1959–1960

Ooi WL, Elston RC, Chen VW, Bailey-Wilson JE and Rothschild H (1986) Increased familial risk for lung cancer. *J Natl Cancer Inst* **76**: 217–222

Osann KE (1991) Lung cancer in women: the importance of smoking, family history of cancer, and medical history of respiratory disease. *Cancer Res* **51**: 4893–4897

Ryberg D, Lindstedt BA, Zienolddiny S and Haugen A (1995). A hereditary genetic marker closely associated with microsatellite instability in lung cancer. *Cancer Res* **55**: 3996–3999

Samet JM, Humble CG and Pathak DR (1986) Personal and family history of respiratory disease and lung cancer risk. *Am Rev Respir Dis* **134**: 466–470

Sellers TA, Ooi WL, Elton RC, Chen VW, Bailey-Wilson JE and Rothschild H (1987) Increased familial risk for non-lung cancer among relatives of lung cancer patients. *Am J Epidemiol* **126**: 237–246

Shaw GL, Falk RT, Pickle LW, Mason TJ and Buffler PA (1991) Lung cancer risk associated with cancer in relatives. *J Clin Epidemiol* **44**: 429–437

Shimizu H and Burns JC (1995) Extraction of nuclear acids: Sample preparation from paraffin-embedded tissues. In *PCR Strategies*. Innis MA, Gelfand DH and Sninsky JJ (eds) pp. 32–38. Academic Press: San Diego

Shridhar V, Siegfried J, Hunt J, Del Mar Alonso M and Smith DI (1994) Genetic instability of microsatellite sequences in many non-small cell lung carcinomas. *Cancer Res* **54**: 2084–2087

Tokuhata GK and Lilienfeld AM (1963) Familial aggregation of lung cancer in humans. *J Natl Cancer Inst* **30**: 289–312

Tsuchiya E, Nakamura Y, Weng SY, Nakagawa K, Tsuchiya S, Sugano H, Kitagawa T (1992) Allelotype of non-small cell lung carcinoma – comparison between loss of heterozygosity in squamous cell carcinoma and adenocarcinoma. *Cancer Res* **52**: 2478–2481

Tsugane S, Watanabe S, Sugimura H, Arimoto H, Shimosato Y and Suemasu K (1987) Smoking, occupation and family history in lung cancer patients under fifty years of age. *Jpn J Clin Oncol* **17**: 309–317

Wooster R, Cleton-Jansen AM, Collins N, Mangion J, Cornelis RS, Cooper CS, Gusterson BA, Ponder BAJ, Von Deimling A, Weistler OD, Cornelisse CJ, Devilee P and Stratton MR (1994) Instability of short tandem repeats (microsatellite) in human cancers. *Nature Genet* **6**: 152–156

World Health Organization (1981) *Histological Typing of Lung Tumors*, 2nd ed. World Health Organization: Geneva

Wu AH, Yu MC, Thomas DC, Pike MC and Henderson BE (1988) Personal and family history of lung disease as risk factors for adenocarcinoma of the lung. *Cancer Res* **48**: 7279–7284

© Cancer Research Campaign 1998